# Increased direct oral anticoagulant use and event rates in non-valvular atrial fibrillation: a nationwide retrospective registry study in Sweden

**DOI:** 10.1136/bmjopen-2025-100960

**Published:** 2025-07-15

**Authors:** Hayder Kadhim, Martin Jansson, Sara Själander, Vilhelm Sjögren, Fredrik Björck, Henrik Renlund, Marie Eriksson, Bo Norrving, Anders Själander

**Affiliations:** 1 Department of Public Health and Clinical Medicine, Umeå University, Umeå, Sweden; 2 Uppsala Clinical Research Center, Uppsala University, Uppsala, Sweden; 3 Department of Statistics, USBE, Umeå University, Umeå, Sweden; 4 Department of Clinical Sciences Lund, Neurology, Skåne University Hospital, Lund University Hospital, Lund, Sweden

**Keywords:** Adult cardiology, Anticoagulation, Cerebral Hemorrhage, Stroke medicine

## Abstract

**Abstract:**

**Rationale:**

The use of direct oral anticoagulants (DOACs) as stroke prophylaxis in patients with non-valvular atrial fibrillation (NVAF) has increased steadily since the introduction in 2011. In Sweden today, more patients are treated with DOACs than with warfarin. However, it is not shown that an increased proportion of DOAC prescriptions correlates to lower event rates of stroke and systemic embolism.

**Objectives:**

This study aims to investigate whether the increased prescription of DOACs in Sweden correlates with lower event rates for all-cause stroke, systemic embolism and bleeding complications, using real-life data for the whole NVAF population.

**Design:**

Nationwide retrospective register study.

**Setting:**

Data were obtained from the Swedish National Patient Registry, covering patients aged 18 years or older with NVAF, between 1 January 2014, and 31 December 2017. Exposure to oral anticoagulants was determined based on pharmaceutical data, calculating treatment duration by the number of pills dispensed and the prescribed daily usage rate. Baseline characteristics and endpoints were collected from hospital administrative registers using International Classification of Diseases, 10th edition (ICD-10) codes.

**Participants:**

All patients with NVAF were identified using ICD-10 codes during the study period. Entry criteria included having a first recorded atrial fibrillation diagnosis after 1 January 2014 or being previously diagnosed with atrial fibrillation before 2014 but still receiving care after this date.

**Outcome measures:**

The outcomes were event rates (per 100 person-years) of ischaemic stroke, systemic embolism, all-cause stroke, major bleeding and intracranial bleeding (including haemorrhagic stroke). Event rates were calculated and compared across the study period using Cox proportional hazard models.

**Results:**

In the total NVAF population, the proportional decrease in event rates (per 100 treatment years) in 2017 compared with 2014 was ischaemic stroke 24% (1.70–1.30), all-cause mortality 4% (9.40–9.00), all-cause stroke 24% (2.10–1.60) and all-cause stroke and systemic embolism 23% (2.20–1.70). During the same time, the proportion of major bleeding and intracranial bleeding rates, including haemorrhagic stroke, also decreased: 5% (2.00–1.90), 6% (0.68–0.64) and 17% (0.30–0.25), respectively. DOACs use increased from 4.1% to 28.3% in the total population and from 22.7% to 60.9% in newly diagnosed patients.

**Conclusions:**

In the initial years following the introduction when DOAC uptake in the population was high, an increasing proportion of Swedish NVAF patients receiving DOACs was accompanied by lower event rates of all-cause stroke and systemic embolism, ischaemic stroke and all-cause mortality, intracranial bleeding and major bleeding, highlighting the improved risk-benefit balance of DOACs in stroke prophylaxis.

**STRENGTHS AND LIMITATIONS OF THIS STUDY**

- Large nationwide registry study: This study utilises comprehensive national health registries with high coverage, ensuring a representative sample of the Swedish non-valvular atrial fibrillation (NVAF) population over four consecutive years.
- Temporal relevance: The study period coincides with the most significant increase in direct oral anticoagulant usage, maximising its potential impact on NVAF outcomes compared with later years, when changes in usage were smaller.
- Dispensation vs adherence: The study design identifies only medication dispensation from pharmacies, without confirming whether patients adhered to the prescribed regimen.
- Potential diagnostic limitations: The accuracy of diagnoses and outcomes coding in the registries may affect the results, with potential misclassification or missing diagnoses, particularly due to the lack of primary healthcare data.

## Introduction

 For the last 50 years, vitamin K antagonists (VKAs), for example, warfarin, have been the standard of care for stroke prophylaxis in non-valvular atrial fibrillation (NVAF) patients.^1^ Warfarin is superior to aspirin, reducing stroke risk by about two-thirds but increasing the risk of haemorrhagic stroke in patients with NVAF.^2^,^4^ The reduction in ischaemic strokes outweighs the bleeding risk,^5^ even in frail patients with frequent falls.^6 7^ However, VKAs are associated with several limitations,^1 8 9^ and there are a range of factors affecting the treatment quality.^10 11^

In pivotal and later observational studies, direct oral anticoagulants (DOACs), including edoxaban, apixaban, dabigatran and rivaroxaban, are non-inferior or superior to warfarin in terms of reduced stroke risk while also carrying a similar or lower risk of bleeding.^12^,^16^ DOACs are now the drugs of choice in patients newly diagnosed with NVAF.^17^

The use of DOACs has steadily increased every year since the introduction in 2011. During the study years, 2014–2017, the uptake of DOACs in the NVAF population was at its peak, and thus, one can assume it had the greatest impact on the group’s outcomes. We aim to study how the rapid rise in DOAC prescriptions has impacted the rates of ischaemic stroke and the prevalence of bleeding complications.

## Methods

Patients with NVAF in Sweden between 1 January 2014 and 31 December 2017 aged 18 or older were identified in the Swedish National Patient Registry (NPR) using the ICD-10 (International Classification of Diseases, 10th edition) code I48. The NPR is a Swedish healthcare administrative registry founded in 1964. The Swedish National Board of Health and Welfare manages the registry, which contains data from both inpatient and outpatient care. The coverage is almost 100%, and the positive predictive value (PPV) of a diagnosis is 85–97% with a PPV of atrial fibrillation of 97%.^18^ Exposure to oral anticoagulation therapy was determined using the Swedish Prescribed Drug Register (SPDR), where the type of medication, date of prescription, dosage and amount are registered by all pharmacies in Sweden.^19^ SPDR includes information on all prescribed and dispensed medications with almost 100% coverage.

The source for stroke diagnoses was Riksstroke, a nationwide register for stroke in Sweden with high data input precision and marginal risk for misclassification. The compatibility rate between the diagnoses entered in Riksstroke and the medical records is about 95%, and the estimated coverage of Riksstroke was 89% in 2019.^20 21^ Date of death was retrieved from the cause of death register (CDR).^22^

Intracranial bleeding was defined as haemorrhagic stroke or traumatic intracranial bleeding. Haemorrhagic stroke includes intracerebral haemorrhage (ICH) and subarachnoid haemorrhage. Major bleeding was defined as bleeding that required inpatient care. Diagnoses were obtained from NPR.

### Statistical methods

A patient’s study entry date was the date of their first NVAF diagnosis unless the diagnosis occurred before 2014, when their entry date was set to 1 January 2014. Patients with their first NVAF diagnosis during the study period were classified as ‘new’. Concomitant disorders were registered using ICD-10 codes from the NPR and oral antithrombotic treatment from prescriptions registered in the SPDR. The treatment duration for each drug was calculated from the total pills dispensed and the daily usage rate. Each drug’s daily usage was predefined (eg, apixaban 2 pills/day and clopidogrel 1 pill/day). The stockpile decreases daily, and when pills run out, the treatment was marked as stopped unless a new dispensation occurred, extending the treatment duration. We monitor dispensations from 6 months prior to the study entry date to determine treatment durations. However, we only consider durations that overlap with each individual’s study entry and exit dates. A dispensation of a different type of oral anticoagulant (OAC) terminates the treatment duration of the previous one.

All patients were followed until the end of the study or time of death. Event rates of the following outcomes were estimated: major bleeding (including intracranial, GI or other), ischaemic stroke, peripheral thromboembolism and myocardial infarction (MI). To avoid over-registering, recurrent events of the same type within 14 days were not recorded. After an event other than death, a patient will be censored for that event but remain in the cohort for other types of events.

Event rates were calculated as the number of events (*n*)/patient time (years) and presented as events per 100 years. We used separate Cox proportional hazard regression models to estimate hazard ratios (HR) for each event type, comparing each year (2015–2017) to the year 2014. For these analyses, each individual has a 365-day clock and a cohort year indicator. A new patient who enters after the first of January in a given year will start their clock at the corresponding day of the year; if they survive, the clock is reset to 0, and the cohort year indicator is updated at the beginning of the next year. Robust SDs are used to account for the fact that individuals can contribute time to more than one cohort year. The proportional hazards assumptions were inspected visually. Statistical analysis was performed using R version 4.4.1. Event rates regarding the cohort per year were unadjusted to age or other outcome risk factors.

### Patient and public involvement statement

Patients were first involved in this research during their contact with healthcare providers, where they were informed that their health data would be stored in national registers and could be used for research purposes. They were given the right to opt out of data storage for any reason. The research questions were developed based on the available health data from national registers, ensuring alignment with public health priorities. However, patients did not directly contribute to shaping the research questions. The study utilised de-identified health data obtained from national registers, ensuring patient privacy.

Patients and the public were not directly engaged in selecting methods or planning the dissemination of results. However, the study findings will be communicated through appropriate scientific and public health channels, ensuring accessibility to relevant stakeholders and communities.

### Ethical approval and consent to participate

The study was approved, and the need for informed consent was waived by the regional ethics committee in Umeå (dnr 2015/142–31). All methods were carried out in accordance with relevant guidelines and regulations. The health data used in the research have been obtained de-identified from the national registers. Patients have the right to deny saving their health data, whatever the reason.

## Results

### Population description

#### Total population

1. In total, registered patients with NVAF on the first of January were 277 278, 288 967, 299 753 and 310 551 for 2014, 2015, 2016 and 2017, respectively. The mean age of included patients was 76.6 years (69.0–84.1), and the female proportion was 42% (table 1). NVAF prevalence was 3.1% in 2017.

**Table 1:** Baseline clinical characteristics and treatment for all patients with NVAF in Sweden in the beginning of years 2014–2017, presented as n (%) if otherwise not stated

	**2014**(*n* = 2 77 278)	**2015**(*n* = 2 88 967)	**2016**(*n* = 2 99 753)	**2017**(*n* = 3 10 551)
Demographic characteristics				
NVAF prevalence (%)	2.88	2.97	3.04	3.11
Remaining from 2014 cohort	100	88.1	78.1	69.3
Age (years), median (Q1–Q3)	76.6 (68.2–84.2)	76.6 (68.5–84.2)	76.6 (68.7–84.2)	76.6 (69.0–84.1)
Female gender	116 192 (41.9)	120 448 (41.7)	124 494 (41.5)	128 195 (41.3)
Prior medical conditions				
Anaemia	35 409 (12.8)	37 779 (13.1)	40 537 (13.5)	43 420 (14)
Cancer	63 460 (22.9)	68 957 (23.9)	74 203 (24.8)	79 619 (25.6)
Chronic obstructive pulmonary disease	23 187 (8.4)	24 327 (8.4)	25 404 (8.5)	26 627 (8.6)
Congestive heart failure	82 369 (29.7)	84 856 (29.4)	87 307 (29.1)	90 248 (29.1)
Diabetes	50 323 (18.1)	53 080 (18.4)	55 513 (18.5)	58 121 (18.7)
Excessive alcohol use	10 362 (3.7)	11 212 (3.9)	12 085 (4)	13 037 (4.2)
Hypertension	169 657 (61.2)	181 552 (62.8)	192 064 (64.1)	202 161 (65.1)
Renal failure	18 961 (6.8)	20 923 (7.2)	23 225 (7.7)	25 742 (8.3)
Ischaemic stroke	45 393 (16.4)	46 876 (16.2)	48 053 (16)	48 625 (15.7)
Stroke or TIA	58 042 (20.9)	60 404 (20.9)	62 297 (20.8)	63 647 (20.5)
TIA	20 631 (7.4)	21 872 (7.6)	22 954 (7.7)	24 076 (7.8)
Vascular disease	62 163 (22.4)	64 467 (22.3)	66 598 (22.2)	68 746 (22.1)
Liver disease	4398 (1.6)	4833 (1.7)	5261 (1.8)	5771 (1.9)
Myocardial infarction	49 875 (18)	51 394 (17.8)	52 673 (17.6)	53 969 (17.4)
CHA_2_DS_2_–VASc score				
Mean (SD)	3.4 (1.76)	3.42 (1.76)	3.43 (1.75)	3.44 (1.75)
0 point men/1 point women	23 313 (8.4)	23 676 (8.2)	24 139 (8.1)	24 566 (7.9)
1 point men/2 points women	30 817 (11.1)	31 521 (10.9)	32 156 (10.7)	32 832 (10.6)
≥2 points men/≥3 points women	223 148 (80.5)	233 770 (80.9)	243 458 (80.5)	253 153 (80.5)
Prior bleeding				
Major bleeding	70 735 (25.5)	76 799 (26.6)	82 892 (27.7)	89 298 (28.8)
Intracranial bleeding	8588 (3.1)	9345 (3.2)	10 164 (3.4)	10 891 (3.5)
Haemorrhagic stroke	4588 (1.7)	4922 (1.7)	5280 (1.8)	5570 (1.8)
Traumatic intracranial bleeding	3490 (1.3)	3842 (1.3)	4292 (1.4)	4626 (1.5)
Gastrointestinal bleeding	4450 (1.6)	4671 (1.6)	4899 (1.6)	5037 (1.6)
Other bleeding	60 982 (22)	66 502 (23)	71 812 (24)	77 500 (25)
Treatment				
DOACs	11 280 (4.1)	29 721 (10.3)	57 479 (19.2)	88 026 (28.3)
Apixaban	1478 (0.53)	11 552 (4)	31 849 (10.6)	55 305 (17.8)
Dabigatran	6171 (2.2)	8879 (3.1)	9582 (3.2)	11 538 (3.7)
Edoxaban	0 (0)	0 (0)	0 (0)	56 (0.018)
Rivaroxaban	3631 (1.3)	9290 (3.2)	16 048 (5.4)	21 127 (6.8)
Warfarin	122 473 (44.2)	122 779 (42.5)	114 278 (38.1)	101 554 (32.7)
Thrombocyte inhibitors	68 411 (24.7)	57 383 (19.9)	47 300 (15.8)	39 682 (12.8)
No treatment	75 114 (27.1)	79 084 (27.4)	80 696 (26.9)	81 289 (26.2)

DOAC, direct oral anticoagulant; NVAF, non-valvular atrial fibrillation.

1. CHA_2_DS_2_–VASc score: Mean (SD) values for 2014–2017 were 3.40 (1.76), 3.42 (1.76), 3.43 (1.75) and 3.44 (1.75), respectively. In 2017, the proportion of patients that had 0 points for men or 1 point for women was 7.9%, 1 point for men or 2 points for women was 10.6%, and 2 points or more for men or 3 points or more for women was 81.6%.
2. The proportion of patients with OAC therapy increased from 48.3% in 2014 to 61% in 2017, with ‘DOACs’ increasing gradually from 4.1% to 28.3% (table 1). The use of apixaban increased gradually from 0.5% in 2014 to 17.8% in 2017, while treatment with dabigatran and rivaroxaban increased from 2.2% to 3.7% and 1.3% to 6.8%, respectively (table 2). Treatment with edoxaban was limited (0–0.02%). The proportion with ‘Warfarin’ decreased from 44.2% to 32.7%, and with ‘thrombocyte inhibitors’ decreased from 24.7% to 12.8%, while ‘no treatment’ slightly decreased (27.1–26.2%).

**Table 2:** Baseline clinical characteristics and treatment for newly diagnosed patients with NVAF in Sweden during 2014–2017, presented as n (%) if otherwise not stated

	**2014**(n=38 226)	**2015**(n=38 329)	**2016**(n=38 697)	**2017**(n=38 120)
Demographic characteristics				
Age	76.3 (68.1–84.2)	76.4 (68.3–84.2)	76.2 (68.6–84.0)	76.2 (687–84.0)
Female gender	15 161 (44.1)	15 238 (44.3)	15 312 (44.5)	15 264 (43.8)
Prior medical conditions				
Anaemia	4754 (12.4)	4903 (12.8)	5154 (13.3)	5149 (13.5)
Cancer	8921 (23.3)	9366 (24.4)	9670 (25)	9945 (26.1)
Chronic obstructive pulmonary disease	3172 (8.3)	3300 (8.6)	3511 (9.1)	3383 (8.9)
Congestive heart failure	8070 (21.1)	8045 (21)	8280 (21.4)	7932 (20.8)
Diabetes	6568 (17.2)	6689 (17.5)	6850 (17.7)	6951 (18.2)
Excessive alcohol use	1367 (3.6)	1438 (3.8)	1549 (4)	1565 (4.1)
Hypertension	22 052 (57.7)	22 507 (58.7)	23 045 (59.6)	22 632 (59.4)
Renal failure	2649 (6.9)	2963 (7.7)	3231 (8.3)	3423 (9)
Ischaemic stroke	5575 (14.6)	5573 (14.5)	5431 (14)	5261 (13.8)
Stroke or TIA	7118 (18.6)	7073 (18.5)	7009 (18.1)	6841 (17.9)
TIA	2320 (6.1)	2357 (6.2)	2434 (6.3)	2378 (6.2)
Vascular disease	8099 (21.2)	8099 (21.1)	8281 (21.4)	8106 (21.3)
Liver disease	645 (1.7)	676 (1.8)	676 (1.7)	727 (1.9)
Myocardial infarction	6430 (16.8)	6418 (16.7)	6541 (16.9)	6445 (16.9)
CHA_2_DS_2_–VASc score				
Mean (SD)	3.26 (1.72)	3.27 (1.71)	3.28 (1.71)	3.28 (1.72)
0 point men/1 point women	3407 (8.9)	3391 (8.8)	3285 (8.5)	3325 (8.7)
1 point men/2 points women	4904 (12.8)	4810 (12.6)	4892 (12.6)	4819 (12.6)
≥ 2 points men/≥3 points women	29 915 (78.3)	30 118 (78.6)	30 520 (78.9)	29 976 (78.6)
Prior bleeding				
Major bleeding	7985 (20.9)	8212 (21.4)	8590 (22.2)	8727 (22.9)
Intracranial bleeding	966 (2.5)	1097 (2.9)	1091 (2.8)	1113 (2.9)
Haemorrhagic stroke	546 (1.4)	593 (1.5)	581 (1.5)	598 (1.6)
Traumatic intracranial bleeding	389 (1)	450 (1.2)	433 (1.1)	464 (1.2)
Gastrointestinal bleeding	589 (1.5)	583 (1.5)	542 (1.4)	571 (1.5)
Other bleeding	6689 (17.5)	6835 (17.8)	7231 (18.7)	7384 (19.4)
Treatment during first follow-up quarter				
DOAC	8677 (22.7)	15 519 (40.6)	20 450 (53.5)	23 215 (60.9)
Warfarin	13 073 (34.2)	8279 (21.6)	4875 (12.6)	3087 (8.1)
Thrombocyte inhibitors	6498 (17.0)	5097 (13.3)	4295 (11.1)	3659 (9.6)
No treatment	10 015 (26.2)	9390 (24.5)	8861 (22.9)	8157 (21.4)

AF, Atrial fibrillation; CHA2DS2–VASc, Congestive heart failure, Hypertension, Age (≥75 years, doubled), Diabetes, Stroke/TIA/thromboembolism (doubled), Vascular disease, Age (65-74 years), and Sex (female); DOAC, direct oral anticoagulant; ICD, International Statistical Classification of Diseases; NVAF, non-valvular atrial fibrillation; TIA, Transient Ischemic Attack.

#### Newly diagnosed patients

1. There were 38 226, 38 319, 38 697 and 38 120 newly diagnosed for 2014, 2015, 2016 and 2017, respectively. The mean age of newly diagnosed patients was 76.2 (68.7–84) years in 2017, and the female proportion was 44.5% (table 2).
2. CHA_2_DS_2_–VASc score: Mean (SD) values for 2014–2017 are 3.26 (1.72), 3.27 (1.71), 3.28 (1.71) and 3.28 (1.75), respectively. In 2017, the proportions of patients were 0 points for men or 1 point for women, 8.7%; 1 point for men or 2 points for women, 2.6%; and 2 points or more for men or 3 points or more for women, 78.6%.
3. The proportion of patients with OAC therapy increased from 56.9% in 2014 to 69.0% in 2017, with ‘DOACs’ increasing from 22.7% to 60.9% while warfarin treatment decreased from 34.2% to 8.1% (table 2). The proportion of patients with only thrombocyte inhibitors or no antithrombotic treatment decreased (17.0–9.6% and 26.2–21.4%, respectively).

### HR and event rates

#### Individual risks

**Figure 1:**
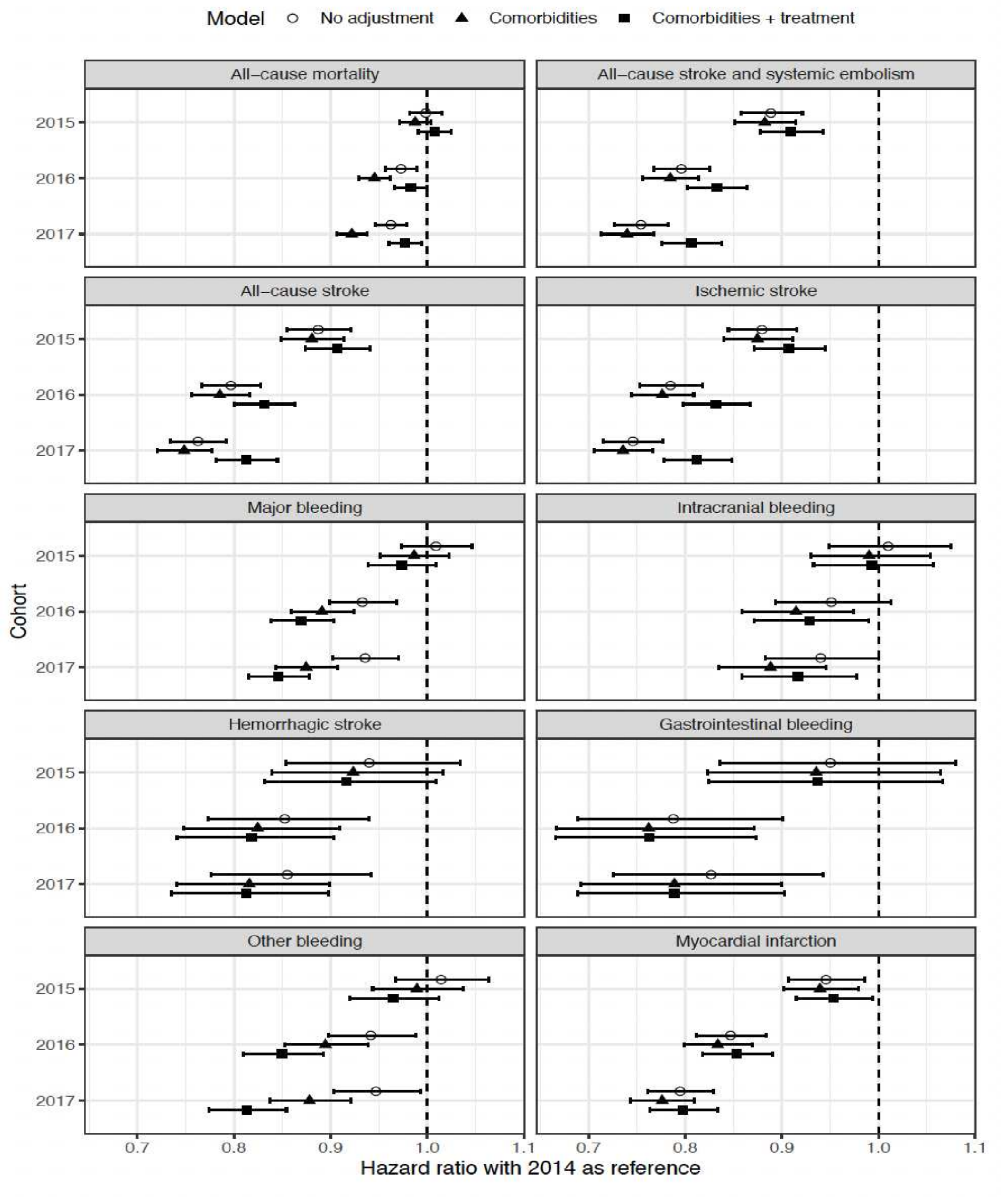
HR of death and other severe outcomes for each cohort year (2015–2017) compared with 2014 in a model adjusted for cohort, age, gender, chronic diseases, alcohol abuse, gastrointestinal bleeding, ICH, stroke or TIA and medication. HR: hazard ratio; ICH: intracranial haemorrhage.

The HR for the total population, comparing each event type for the years 2015, 2016, and 2017 against the baseline year 2014, is presented in figure 1. The findings are adjusted for cohort characteristics, age, gender, chronic diseases, gastrointestinal bleeding (GI bleeding), ICH, alcohol abuse, stroke or TIA and medication use. The results show a significant decrease in HR for ischaemic stroke, all-cause mortality, all-cause stroke, all-cause stroke and systemic embolism, major bleeding, ICH and MI in 2017 compared with 2014.

#### Population event rates

**Figure 2:**
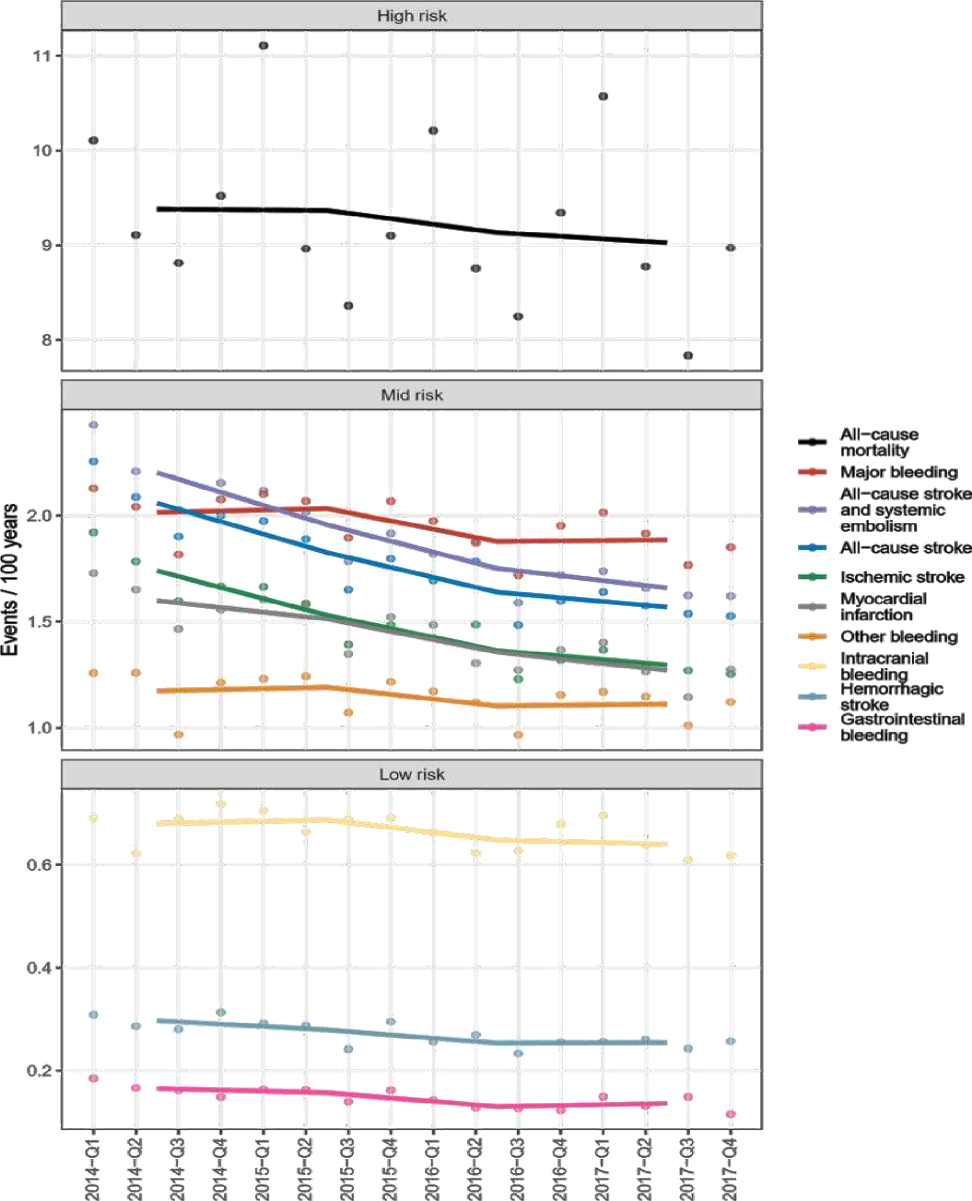
Event rate over time. The dots are averages over the yearly quarters. The lines are between the yearly averages. Grouping into risk strata for ease of plotting only. Please note that we made technical adjustments to the *y*-axis of the figure, such as skipping values 3–7 and reducing the spacing between grades in the high-risk category, to fit the figure onto a single page.

Event rates (per 100 treatment years) of ischaemic stroke, all-cause mortality, all-cause stroke and all-cause stroke and systemic embolism decreased from 1.70 to 1.30, 9.40 to 9.00, 2.10 to 1.60 and 2.20 to 1.70, respectively. The rate of major bleeding, GI bleeding, intracranial bleeding, haemorrhagic stroke and other bleeding was stable or slightly decreased during the study period from 2.00 to 1.90, 0.17 to 0.14, 0.68 to 0.64, 0.30 to 0.25 and 1.20 to 1.10 (table 3). Event rate over time presented graphically in (figure 2).

**Table 3:** Event count by year

	2024		2015		2016		2017	
Endpoint	Events	Rate	Events	Rate	Events	Rate	Events	Rate
All-cause mortality	26 545	9.40	27 533	9.40	27 910	9.10	28 380	9.00
All-cause stroke and systemic embolism	6228	2.20	5750	2.00	5350	1.80	5217	1.70
All-cause stroke	5824	2.10	5366	1.80	5008	1.60	4933	1.60
Ischaemic stroke	4922	1.70	4497	1.50	4168	1.40	4076	1.30
Major bleeding	5699	2.00	5973	2.00	5739	1.90	5927	1.90
Intracranial bleeding	1925	0.68	2020	0.69	1981	0.65	2013	0.64
Haemorrhagic stroke	841	0.30	821	0.28	775	0.25	800	0.25
Gastrointestinal bleeding	468	0.17	462	0.16	398	0.13	429	0.14
Other bleeding	3321	1.20	3499	1.20	3371	1.10	3495	1.10
Myocardial infarction	4524	1.60	4445	1.50	4146	1.40	3995	1.30

Rate is events/100 years. The patient times for the years 2014–2017 in 100-year units are 2829.5, 2939.1, 3055.6 and 3144.1, respectively.

## Discussion

This large Swedish nationwide retrospective register study, consisting of 310 551 NVAF patients in January 2017, shows a decreasing rate of all-cause stroke or systemic embolism in the total NVAF population. The number of NVAF patients corresponds to a prevalence of 3.1%, which is within the international upper limit range.^23^ Furthermore, there are significant rate reductions of ischaemic stroke and MI between 2014 and 2017.

In parallel with these rate reductions, there was also an increase in DOAC use in NVAF patients, from 4.1% in 2014 to 28.3% in 2017, and a decrease in antiplatelet treatment from 24.7% to 12.8% in 2017. This reflects the gradual establishment of DOAC as the drug of first choice in NVAF patients and the wide awareness about the guideline recommendations in the past few years. We believe that it is during this period that DOAC increases the most and thus most likely contributes to the NVAF population’s outcome more than in later years, when the yearly change in the proportion of NOAC patients is much smaller.

In a retrospective study in England, they noticed lower AF-related stroke incidence by 11.3% in response to increased use of DOACs.^24^ This study is difficult to compare with ours due to essential differences in study design, lack of background information and different trends in OAC use; for example, it showed 16% more warfarin use over time, in contrast to ours which showed a 26% decrease.

A previous Swedish study during 2011–2013 has noticed increased use of OAC accompanied by a lower rate of ischaemic stroke in a limited study population.^25^ We can here confirm these findings in a large nationwide study. About two-thirds of the NVAF population in 2017 had either warfarin or DOAC as stroke prophylaxis, almost the same proportion as seen in another study performed during this period.^26^

Also, one could assume that the significant increase in DOAC treatment would carry a higher event rate of major bleeding events among NVAF patients. Fortunately, this was not the case. Instead, we could see decreasing rates of intracranial bleeding, GI bleeding and haemorrhagic stroke comparing the first and the last study periods. This could indicate the safety of the wide use of DOACs in NVAF patients. However, another explanation for the stable trend of bleeding rates in the total NVAF population could be a decreased patient proportion with Warfarin treatment (from 44.2% to 32.7%) since Warfarin carries a higher risk of intracranial bleeding compared with DOACs, especially in patients with previous intracranial bleeding.^27 28^ The yearly decrease in ischaemic stroke rates since the introduction of DOACs possibly reflects their effectiveness as stroke prophylaxis in the real-world setting. We have not compared the effectiveness between DOAC and warfarin. However, we have seen that the proportion of patients receiving OAC is increasing. Simultaneously, DOAC is increasing at the expense of warfarin, with a dramatic decrease in thrombocyte inhibitor use as monotherapy. Meanwhile, we continue to have good stroke protection with likely a lower bleeding risk per treated patient with DOACs, which leads to an overall improvement for the entire NVAF group.

These findings support previous studies which show that DOACs are at least similarly effective compared with warfarin.^12 13 15 16^ A large North American retrospective study showed increased ischaemic stroke incidence in patients treated with DOACs compared with warfarin.^29^ The authors state that a possible reason for this discrepancy could be lower compliance in patients treated with DOACs in this setting.

In this register study, there was a large number of NVAF patients without adequate stroke prophylaxis (39% and 31%) of the total NVAF and newly diagnosed population, respectively, in 2017. These patients were either not treated at all or had treatment that was not evidence-based as stroke prophylaxis, that is, thrombocyte aggregation inhibitors.

In 2014, 24.7% of the NVAF patients were treated with only thrombocyte aggregation inhibitors, with a reduction to 12.8% in 2017. This dramatic decrease occurred within a few years, indicating improved adherence to NVAF management guidelines. Nevertheless, suboptimal treatment in NVAF patients is a serious concern. Previous studies have shown that areas with lower OAC treatment coverage have higher stroke incidence.^2630^,^32^

This is a large nationwide register study including all patients with NVAF diagnoses during 4 consecutive years, representing the Swedish NVAF population. The data were retrieved from well-validated national health registries with high coverage.

The design of this study does not identify whether medications were taken as prescribed. The study design can only establish that the medication has been dispensed from the pharmacy. The results may be affected by the accuracy of diagnoses and outcomes coding in the register. There are probably diagnoses missing since there is no primary healthcare (PHC) data. We could also have missed up to 11% of acute stroke diagnoses due to incomplete coverage of Riksstroke, which might lead to underestimated ischaemic stroke event rates. However, we do not believe that these should be unevenly distributed in the population and that Riksstroke is a better option than using the NPR for this specific outcome. For all other outcomes, the NPR is the source.

Furthermore, diagnosis codes may have been incorrectly applied or not indicative of disease. There could have been a shortage of atrial fibrillation and chronic diagnoses registered in PHC before specialist referral. A previous study has shown that 12% of patients have an atrial fibrillation diagnosis in PHC only, but only a marginal effect on CHA_2_DS_2_–VASc score when including PHC diagnoses.^33^

## Conclusions

During the early years following the introduction of direct oral DOACs, there was a significant shift in anticoagulant treatment patterns for patients with NVAF in Sweden. The proportion of patients treated with anticoagulants increased from approximately 48% to 60%, driven by a marked rise in DOAC use and a corresponding decline in warfarin and thrombocyte inhibitor use. At the same time, there were reduced rates of all-cause stroke, systemic embolism, ischaemic stroke and all-cause mortality, accompanied by a simultaneous decrease in the incidence of intracranial bleeding and major bleeding complications. These findings highlight an improved risk-benefit balance in stroke prophylaxis for atrial fibrillation, emphasising the positive impact of expanded DOAC adoption in clinical practice.

## Data Availability

Data are available upon reasonable request.
