# Supplementary material for: Increased direct oral anticoagulant use and event rates in non-valvular atrial fibrillation: a nationwide retrospective registry study in Sweden
Source: BMJ Open. 2025 Jul 15;15(7):e100960. doi: 10.1136/bmjopen-2025-100960 (PMC12265802; doi:10.1136/bmjopen-2025-100960)
Supplement: Supplementary file 1 [file bmjopen-2025-100960.reviewer_comments.pdf]

PEER REVIEW HISTORY

BMJ Open publishes all reviews undertaken for accepted manuscripts. Reviewers are asked to complete a checklist review form and are provided with free text boxes to elaborate on their assessment. These free text comments are reproduced below.

ARTICLE DETAILS

Title (Provisional)

Increased Direct Oral Anticoagulant Use and Event Rates in Non-Valvular Atrial Fibrillation: A Nationwide Retrospective Registry Study in Sweden

Authors

Kadhim, Hayder; Sjögren, Vilhelm; Björck, Fredrik; Jansson, Martin; Själander, Sara; Renlund, Henrik; Eriksson, Marie; Norrving, Bo; Själander, Anders

---

VERSION 1 - REVIEW

**Reviewer**	**1**
**Name**	**Kelson, Zoe**
**Affiliation**	**University of Exeter, Mathematics**
**Date**	**07-Apr-2025**
**COI**	**None**

This study aims to investigate whether the increased prescription of direct oral anticoagulants (DOACs) in Sweden correlates with lower event rates of all -cause stroke, systemic embolism, and bleeding complications in real -life data for the whole non -valvular atrial fibrillation (NVAF) population.

Reviewer comments:

"Data was obtained from the Swedish national patient registry, covering patients aged 18 years or older with NVAF, between January 1, 2014, and December 31, 2017"

and

"The coverage is almost 100% and the positive predictive value (PPV) of a diagnosis is 85 – 9 7 % with a PPV of atrial fibrillation of 97% (18)."

The authors have utilised a representative dataset for analysis in this study.

"Exposure to oral anticoagulation therapy was determined using the Swedish prescribed drug register (SPDR), where type of medication, date of prescription, dosage and amount are registered by the pharmacies "

Can the authors please further comment on the accuracy of this data?

"Baseline characteristics and endpoints were collected from hospital administrative registers using ICD -10 codes"

Can the authors please confirm if there is any missing covariate data?

"A patient's study entry date was the date of their first NVAF diagnosis, unless the diagnosis occurred before 2014 when their entry date was set to January 1, 2014."

Can the authors please consider conducting a sensitivity analysis on this treatment of diagnoses before 2014?

"Event rates were calculated as the number of events (n)/patient time (years) and presented as events per 100 years. We used separate Cox proportional hazard regression models to estimate hazard ratios (HR) for each event type comparing each year (2015, 2016, 2017) to the year 2014."

and

"Proportional hazards assumptions were inspected visually. Statistical analysis was performed using R version 4.4.1. Event rates regarding cohort per year were unadjusted to age or other outcome risk factor"

Appropriate modelling methods have been applied by the authors.

Can the authors please consider exploring adjusted event-rates?

Alternatively/additionally, can the authors please examine event rates stratified by age, gender and other risk factors?

"Temporal Relevance : The study period coincides with the most significant increase in DOAC usage, maximizing its potential impact on NVAF outcomes compared to later years when changes in usage were smaller."

Given the authors use data between 2014 and 2017, can they please further assess whether study outcomes can be considered to be up to date and relevant today?

"Dispensation vs. Adherence : The study design identifies only medication dispensation from pharmacies, without confirming whether patients adhered to the prescribed regimen. • Potential Diagnostic Limitations: The accuracy of diagnoses and outcomes coding in the registries may affect the results, with potential misclassification or missing diagnoses, particularly due to the lack of primary health care data" [strengths and limitations]

A discussion on the study limitations is provided by the authors.

Thanks for providing a copy of the STROBE checklist.

**Reviewer**	**2**
**Name**	**Hemels, Martin**
**Affiliation**	**Rijnstate, Arnhem, Department of Cardiology**

**Date**	**04-May-2025**
**COI**	**None**

Well written article regarding an actual current topic. The results and conclusions are clear, limitations of this retrospective study addressed.

VERSION 1 - AUTHOR RESPONSE

Reviewer: 1

Prof. Zoe Kelson, University of Exeter

Comments to the Author:

This study aims to investigate whether the increased prescription of direct oral anticoagulants (DOACs) in Sweden correlates with lower event rates of all -cause stroke, systemic embolism, and bleeding complications in real -life data for the whole non -valvular atrial fibrillation (NVAF) population.

Reviewer comments:

1- "Data was obtained from the Swedish national patient registry, covering patients aged 18 years or older with NVAF, between January 1, 2014, and December 31, 2017"
and
"The coverage is almost 100% and the positive predictive value (PPV) of a diagnosis is 85 – 9 7 % with a PPV of atrial fibrillation of 97% (18)."
The authors have utilised a representative dataset for analysis in this study.

➔ Thank you for this. We also believe this is a unique population-based dataset.

2- "Exposure to oral anticoagulation therapy was determined using the Swedish prescribed drug register (SPDR), where type of medication, date of prescription, dosage and amount are registered by the pharmacies "
Can the authors please further comment on the accuracy of this data?

➔ Thank you for your valuable comment. The Swedish Prescribed Drug Register (SPDR) is a highly reliable national database that includes information on all prescribed

and dispensed medications in Sweden, with almost 100% coverage. The data entry into the SPDR is automated and based on electronic records from pharmacies, which minimizes the risk of manual errors and eliminates the potential for bias or false input. Therefore, we consider the accuracy of the exposure data obtained from the SPDR to be very high.

To clarify, we add in the methods section: *" Exposure to oral anticoagulation therapy was determined using the Swedish prescribed drug register (SPDR), where type of medication, date of prescription, dosage and amount are registered by all pharmacies in Sweden(19). SPDR includes information on all prescribed and dispensed medications with almost 100% coverage. "*

3- "Baseline characteristics and endpoints were collected from hospital administrative registers using ICD -10 codes"
Can the authors please confirm if there is any missing covariate data?

➔ All baseline characteristics and endpoint diagnoses were obtained from the Swedish National Patient Register, except for stroke diagnoses, which were sourced from the Swedish Stroke Register (Riksstroke). We mentioned in section (Methods) page 4 the following:

"*The NPR is a Swedish health care administrative registry founded in 1964. The registry is managed by the Swedish National Board of Health and Welfare and contains data from both inpatient and outpatient care. The coverage is almost 100% and the positive predictive value (PPV) of a diagnosis is 85–97% with a PPV of atrial fibrillation of 97%"*

And on the same page also:

"*The source for stroke diagnoses was Riksstroke, a nationwide register for stroke in Sweden with high data input precision with marginal risk for misclassification. The compatibility rate between the diagnoses entered in Riksstroke and the medical records is about 95% and the estimated coverage of Riksstroke was 89% in 2019*"

Since the NPR is a national register containing ICD-10 codes used in clinical practice, there will be diagnoses left out by the clinician when considered less important for the current visit. This is a limitation shared by all retrospective register studies. However, as stated above, clinically significant diagnoses such as myocardial infarct or gastrointestinal bleeding are less likely to be missing. *No changes made in the manuscript.*

4- "A patient's study entry date was the date of their first NVAF diagnosis, unless the diagnosis occurred before 2014 when their entry date was set to January 1, 2014." Can the authors please consider conducting a sensitivity analysis on this treatment of diagnoses <u>before 2014</u>?

➔ The study aims to cover all patients with NVAF in Sweden from 2014 until 2017, with no exclusions. Therefore, all patients that were alive on January 1st 2014 were included. From that date, newly diagnosed NVAF-patients were entered and deceased patients removed continuously until the study end. We also separately report a cohort consisting of only newly diagnosed NVAF patients from 2014 and onwards, which we believe would match the sensitivity analysis you are asking for. *No changes made in the manuscript.*

5- "Event rates were calculated as the number of events (n)/patient time (years) and presented as events per 100 years. We used separate Cox proportional hazard regression models to estimate hazard ratios (HR) for each event type comparing each year (2015, 2016, 2017) to the year 2014."
and
"Proportional hazards assumptions were inspected visually. Statistical analysis was performed using R version 4.4.1. Event rates regarding cohort per year were unadjusted to age or other outcome risk factor"
Appropriate modelling methods have been applied by the authors.

Can the authors please consider exploring adjusted event-rates?

Alternatively/additionally, can the authors please examine event rates stratified by age, gender and other risk factors?

➔ Excellent question. We agree that adjusted or stratified event rates can be informative in certain contexts. However, event rates are inherently population-specific and serve primarily a descriptive purpose in our study. Our intent with presenting unadjusted event rates per 100 patient-years was to provide a straightforward overview of the crude burden of events over time.

To assess differences across years while accounting for potential confounders such as age, gender, and other outcome-related risk factors, we applied Cox proportional hazards models. These models estimate hazard ratio (HR), which we believe are more appropriate for comparative purposes and better suited to answering our study questions. In this way, the potential influence of underlying risk factor distributions is addressed within the modeling framework, rather than through stratification of descriptive rates.We hope this clarifies our analytical approach. *No changes made in the manuscript.*

6- "Temporal Relevance : The study period coincides with the most significant increase in DOAC usage, maximizing its potential impact on NVAF outcomes compared to later years when changes in usage were smaller."
Given the authors use data between 2014 and 2017, can they please further assess whether study outcomes can be considered to be up to date and relevant today?

➔ Fortunately, the treatment of patients with atrial fibrillation have improved in general over time, with better outcome in recent years. This can also be seen in our results, where the risk of stroke is decreasing during the study period even when OAC-treatment is adjusted for. To be able to measure the contribution of DOAC to this effect, there need to be a shift in treatment proportion over time. This shift was greatest during the years included in this study, making it ideal for showing this. Nowadays,

almost all NVAF patients are on DOACs, with little difference between years. Hence it would be much harder to estimate the positive effect of DOAC with more recent data. *No changes made in the manuscript.*

7- "Dispensation vs. Adherence : The study design identifies only medication dispensation from pharmacies, without confirming whether patients adhered to the prescribed regimen. • Potential Diagnostic Limitations: The accuracy of diagnoses and outcomes coding in the registries may affect the results, with potential misclassification or missing diagnoses, particularly due to the lack of primary health care data" [strengths and limitations]
A discussion on the study limitations is provided by the authors.

8- Thanks for providing a copy of the STROBE checklist.

Reviewer: 2
Dr. Martin Hemels, Rijnstate, Arnhem, Radboud University Medical Center
Comments to the Author:
Well written article regarding an actual current topic. The results and conclusions are clear, limitations of this retrospective study addressed.

➔ Thank you for the positive and encouraging feedback. We appreciate your recognition of the manuscript's clarity and the handling of study limitations. *No changes made in the manuscript.*

----

1. You have stated that you have used AI in preparation of your manuscript. As per our policy on AI use, please provide a transparent description of its use in your manuscript, including:

• What AI technology was used (the name of the technology)

→ ChatGPT (OpenAI)

• Why this AI technology was used (the reason for its use)

→We used AI to assist in improving the clarity and conciseness of a certain portion of the manuscript. The aim was to refine the language by making point-form summaries of already written text, without altering the scientific content, ideas, or interpretation.

• How the AI technology was used (what the task of the technology was)

→The AI was employed solely to rephrase and condense text that had already been drafted by the authors. This included transforming a section into a more structured, concise format (e.g., bullet points or clearer phrasing) while preserving the original meaning. No new content, analysis, or interpretation was generated by the AI.

• Consider including a summary of the input, output, and the way in which the AI output was reviewed on the part of the authors as supplementary files or additional information for the editor to review. The editor may ask for more information and/or for information to be added to the content for internal use and/or for publication.

→Input:

Strengths and Limitations

This is a large nationwide register study including all patients with NVAF diagnoses during four consecutive years, representing the Swedish NVAF population. The data was retrieved from well validated national health registries with high coverage. We believe that it is during this period that DOAC increases the most, and thus most likely

contributes to NVAF population´s outcome more than in later years, when the yearly change in the proportion of NOAC patients is much smaller.
However, since this is a register study, residual confounding may bias the results. Hence it is not possible to establish causality between OAC treatment and outcome. Also, the design of this study does not identify whether medications were taken as prescribed. The study design can only establish that the medication has been dispensed from the pharmacy. The results may be affected by the accuracy of diagnoses and outcomes coding in the register. There are probably diagnoses missing since there is no primary health care data. However, we do not believe that these should be unevenly distributed in the population.

Output:

Strengths and Limitations

-**Large Nationwide Registry Study**: This study utilizes comprehensive national health registries with high coverage, ensuring a representative sample of the Swedish NVAF population over four consecutive years.
-**Temporal Relevance**: The study period coincides with the most significant increase in DOAC usage, maximizing its potential impact on NVAF outcomes compared to later years when changes in usage were smaller.
-**Dispensation vs. Adherence**: The study design identifies only medication dispensation from pharmacies, without confirming whether patients adhered to the prescribed regimen.
-**Potential Diagnostic Limitations**: The accuracy of diagnoses and outcomes coding in the registries may affect the results, with potential misclassification or missing diagnoses, particularly due to the lack of primary health care data.

2. Please add a statement on your ethics approval to the methods section.

→ We added to the methods section: *"The study was approved and the need for informed consent waived by the regional ethics committee in Umea (dnr 2015/142-31). All methods were carried out in accordance with relevant guidelines and regulations. The health data used in the research have been obtained de-identified from the national registers. Patients have the right to deny saving their health data whatever the reason".*

VERSION 2 - REVIEW

**Reviewer**	**1**
**Name**	**Kelson, Zoe**
**Affiliation**	**University of Exeter, Mathematics**
**Date**	**16-Jun-2025**
**COI**	

Thanks to the authors for their responses and clarifications. I have no further comments.
